# Parental Experiences of Grief after Pregnancy Loss: systematic review of qualitative studies

**DOI:** 10.1192/j.eurpsy.2024.500

**Published:** 2024-08-27

**Authors:** M. R. Duarte, A. Torres, P. S. Carvalho

**Affiliations:** ^1^Departamento de Psicologia e Educação, Universidade da Beira Interior, Covilhã; ^2^CINTESIS@RISE, Aveiro, Portugal

## Abstract

**Introduction:**

Gestational Loss represents a set of abrupt and unexpected losses throughout pregnancy or after childbirth. Every year, around two million babies die after 28 weeks of gestation, with between 14% and 20% of all pregnancies ending in loss. In most situations, pregnancy loss occurs in a pregnancy without signs of risk or irregularities, something that increases the shock and suffering felt by parents.

**Objectives:**

The present study aims to understand the relationship between pregnancy loss and parents’ grief experiences after spontaneous abortion, stillbirth or neonatal death with qualitative evidence.

**Methods:**

This review followed the principles of PRISMA, and the search was carried out in the Web of Science and Scopus databases, aiming to find relevant articles about parental grief experiences resulting from pregnancy loss, published between 2012 and 2022. After research and analysis Of the studies, 15 qualitative studies were included.

**Results:**

The pain and sadness when experiencing the loss of a child was a common point in all the studies found. In this review, the majority of men revealed a duality in wanting to protect, physically and emotionally, their partner, while experiencing their own grief, something that led to the internalization of their emotions and the minimization of their pain. Grieving fathers and mothers report experiencing this process alone, describing the difficulty in expressing what they feel due to the lack of recognition of the loss. It was found that confrontation with other pregnant women leads bereaved parents to reveal jealousy and shame, as well as feelings of guilt. The farewell rituals, the process of writing and talking about their experience helped the women to not feel so alone and to find a purpose: to transform their pain and help other grieving mothers. Fathers and mothers who experienced pregnancy loss stated that the death of their child provided change and growth.

**Conclusions:**

After Pregnancy Loss, adapting to the new reality is extremely painful, despite the work of mourning being necessary and crucial. This process is a search to integrate and accept the reality of the loss of the baby in a way that has meaning for the mother and father, it is the adaptation to a world without the lost child and to a relationship that had been built during the gestation period, which was violently broken. It is necessary for health professionals to be present and available to address these parents’ fears, provide advice and support.

**Disclosure of Interest:**

None Declared

